# Environmental determinants of neurotoxicity: role of heavy metals in neurological disorders

**DOI:** 10.3389/fneur.2026.1867856

**Published:** 2026-08-14

**Authors:** Snehashis Mandal, Priti Dipa, Neha, Dhrita Chatterjee, Kousik Maparu

**Affiliations:** 1 Department of Pharmacology, ISF College of Pharmacy, Moga, Punjab, India; 2 Department of Pharmacy Practice, ISF College of Pharmacy, Moga, Punjab, India; 3 Department of Pharmacology, Calcutta Institute of Pharmaceutical Technology & Allied Health Sciences, Howrah, West Bengal, India

**Keywords:** environmental neurotoxicity, exposome, gut-brain axis, neurodegeneration, neurodevelopmental disorders, oxidative stress

## Abstract

Neurodevelopmental and neurodegenerative disorders are related disorders lying on a spectrum of neural dysfunction with overlapping molecular and cellular mechanisms. Early-life diseases like autism spectrum disorder and attention-deficit/hyperactivity disorder are the result of disturbed neurodevelopment, while late-onset diseases such as Alzheimer’s disease and Parkinson’s disease are defined by progressive neuronal loss and loss of function. Emerging evidence shows that environmental exposures are important, modifiable factors that affect brain health throughout the lifespan. Factors such as air pollution, heavy metals, pesticides, and endocrine-disrupting chemicals act in combination with genetic susceptibility to disrupt neurogenesis, synaptic plasticity, and neuro-immune signaling. These exposures cause persistent epigenetic modifications, oxidative stress, mitochondrial dysfunction, and chronic neuro-inflammation, linking early developmental insults to neurodegenerative processes later in life. The concepts of the exposome and the developmental origins of health and disease provide additional support for the cumulative, lifelong impact of environmental interactions. Mechanistically, the recurrent process of neuronal damage is caused by impaired proteostasis, microglial stimulation, and blood–brain barrier dysfunction. Furthermore, the gut-brain axis is a hyperactive system in which immune responses and microbial metabolites trigger neurobiological responses to external stimuli. This review integrates multidisciplinary evidence to elucidate the mechanism and emphasizes environmental risk mitigation and translational strategies to reduce disease burden and promote lifelong brain resilience.

## Graphical abstract

**Figure:**
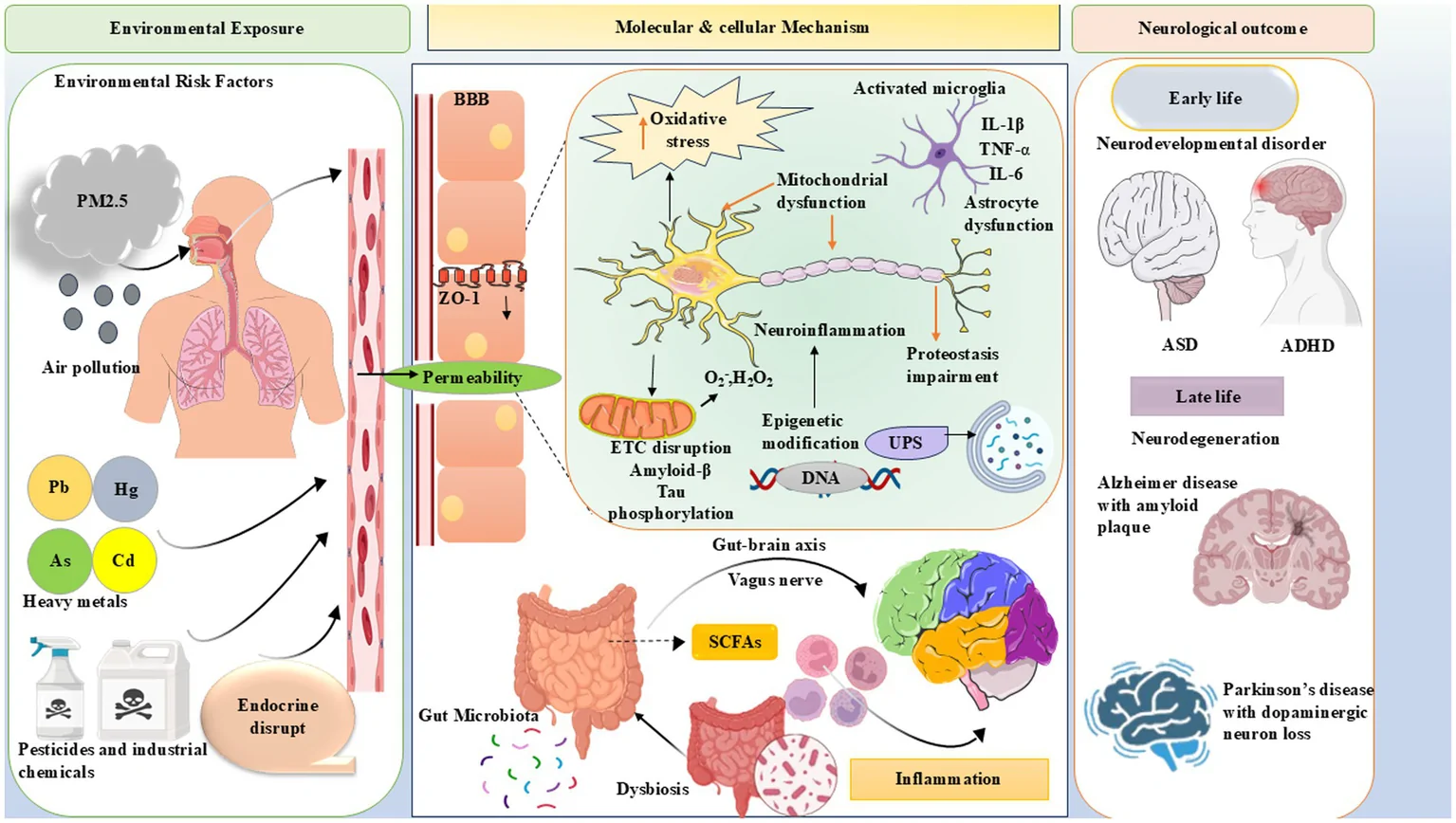

## 1 Introduction

Neurodevelopmental and neurodegenerative disorders involve overlapping biological processes that likely place them at opposite ends of a single dysfunctional spectrum. Early in life, neurodevelopmental disorders such as intellectual disability, attention-deficit/hyperactivity disorder (ADHD), and autism spectrum disorder (ASD) manifest as deficits in behavior, cognition, and adaptive functioning. Neurodegenerative diseases, including Alzheimer’s disease (AD), Parkinson’s disease (PD), and amyotrophic lateral sclerosis (ALS), are characterized by a progressive loss of neurons and function (1). These diseases primarily affect older individuals. Long-term impairment of neural networks, which is the basis for significant disability, reduced quality of life, and increased mortality, is common for both classes of disorders. Over 3.4 billion people around the world are estimated to suffer from neurological disorders, both neurodevelopmental and neurodegenerative disorders (2). These conditions are important causes of a significant portion of the global burden of disease and disability-adjusted life years (DALYs). Neurodegenerative diseases are contributors to disproportionate morbidity and care dependency in the older population, and similarly, neurodevelopmental disorders alone account for almost 80 million years of healthy life lost in 2021 (3). This great and growing burden is due to demographic changes such as aging and population growth, better survival of acute conditions, and increased awareness of previously underdiagnosed disorders. Age and genetic predisposition remain major factors in nervous system disease, but new research reveals the importance of environmental factors as major, often changeable, pathogenic contributors throughout life (4). Evidence for the effects of common environmental exposures, including air pollution, heavy metals, pesticides, and endocrine-disrupting chemicals, on the developing brain and neuronal health in later life has challenged traditional models of etiology that separate neurodevelopmental and neurodegenerative conditions (5). Early-life and late-life neurological pathology are not clearly separated because environmental factors interact with genetic susceptibility to affect neuronal survival, synaptic connectivity, and neuro-immune signaling. These links have good biological plausibility, supported by mechanistic studies. ASD risk in children has been associated with early-life exposure to particulate matter, suggesting that environmental factors interfere with neurogenesis and synaptic plasticity (6). Protein mis-folding, chronic neuro-inflammation, oxidative stress, and mitochondrial dysfunction, processes vital to the pathophysiology of AD and PD, have also been associated with long-term exposure to air pollutants and other neuro-toxicants in adulthood. Furthermore, theories such as the neurodevelopmental origins of neurodegeneration suggest that environmental insults during critical developmental periods may permanently affect cells and their epigenetics, and program a lifetime susceptibility to neurodegenerative processes (7). The goals of this review are to identify environmental risk factors implicated throughout the lifespan, understand common molecular and cellular mechanisms that link early-life exposures to late-onset neural degeneration, highlight disease-specific and cross-cutting pathways, and delineate translational and preventive strategies based on this integrative perspective (8). This review covers disciplines that have been studied separately in the past and provides a coherent framework for understanding how environmental factors can influence brain health from development through aging.

## 2 Environmental exposures across the lifespan: a unified framework

Environmental exposures influence the risk trajectories of both neurodevelopmental and neurodegenerative disorders by affecting nervous system health throughout life, from fetal development to old age. To understand how early-life and lifetime insults interact with genetic predispositions to influence disease susceptibility, a unifying framework encompassing this continuum is needed (9). Since neurons can proliferate, differentiate, and form synapses rapidly during pregnancy, this is one of the most susceptible periods of time for environmental influences on brain development. Adverse maternal environmental factors, including stress, toxicants, air pollutants, and endocrine-disruption chemicals, modulate gene activity through DNA methylation without altering the underlying DNA sequence, thereby disrupting epigenetic reprogramming and impairing critical neurodevelopment processes (10). All of these changes have been linked to impaired neurogenesis, altered synaptic plasticity, and an increased risk of neurological conditions in children, as well as attention-deficit/hyperactivity disorder (ADHD) and autism spectrum disorder (ASD) (11). Prenatal exposures can program lifelong susceptibility to neurological dysfunction while simultaneously altering the structural and functional development of the brain (12). This idea is embodied in the fetal origins and developmental origins of health and disease (DOHaD) hypotheses, which contend that environmental stimuli during pregnancy have long-lasting effects on metabolism and physiology, thereby influencing disease risk in later life (13). Although the developing brain is particularly vulnerable to environmental perturbations, exposures during adolescence and adulthood also disrupt cellular homeostasis, induce oxidative stress, and modulate neuro-immune responses, thereby influencing overall neurological health. Synaptic refinement and myelination during adolescence increase susceptibility to air pollution, chemical mixtures, and lifestyle factors, which in turn alter neural circuitry and cognitive function (14). It is proposed that these critical developmental periods extend beyond the perinatal period, based on epidemiological studies linking cumulative exposure to ambient air pollutants during childhood and adolescence to changes in metabolic pathways and biomarkers associated with later neurological outcomes (15). The developing brain is particularly vulnerable to environmental disruptions; however, exposures during adolescence and adulthood also affect neuro-immune responses, oxidative stress, and cellular homeostasis, all of which have implications for neurological health. Adolescence is a period of continued synaptic refinement and myelination, and adolescents are vulnerable to a host of environmental contaminants, chemical mixtures, and lifestyle choices that can affect neural circuitry and cognitive function. Epidemiological evidence linking cumulative exposure to ambient air pollutants during childhood and adolescence with alteration in metabolic pathways and neurological biomarkers supports the hypothesis that the critical window of neurodevelopment extends beyond the perinatal period. The impact of cumulative environmental exposures worsens with age due to declining physiological reserves, reduced anti-oxidative defense mechanisms, and increased blood–brain barrier permeability (16). Importantly, environmentally mediated neuropathology may be cumulative and progressive, and neurological effects of environmental exposures can manifest long after the initial environmental insult. Prenatal and early exposures can trigger permanent epigenetic changes, immune programming and disturbed organization of synapses, without causing clinical symptoms until the later stages of development. Pollutants and toxicants that promote oxidative stress, neuro-immune sensitization and mitochondrial dysfunction may further increase during adolescence and adulthood and contribute to a greater vulnerability to age-related neurodegenerative diseases. There are parallels with inflammatory disorders characterized by ongoing pathological immune responses and delayed temporal relationships, which highlight the need for life span approaches to neurological risk. Neurodegenerative risk is determined by the interplay between aging biological processes and lifetime exposure to environmental toxicants, as summarized by the exposome concept, which encompasses all internal and external exposures throughout life. Gene–environment interactions have a substantial effect on a person’s lifetime vulnerability to environmental risk factors (17). Interactions between folate metabolism and genetic polymorphisms, as observed in neuronal tube defects, determine the extent to which environmental factors influence developmental outcomes, indicating that specific genetic variants may modulate the impact of environmental exposures on metabolic pathways, detoxification capacity, and epigenetic regulation (18). In complex neurological disorders, such as schizophrenia and ASD, environmental exposures and their temporal, dose, and duration influence the genetic backgrounds to shape the development and resiliency of neural circuits. Genetic predispositions are insufficient to explain disease risk (19).

## 3 Major environmental risk factors implicated in brain disorders

### 3.1 Heavy metals and metalloids

**Figure 1:**
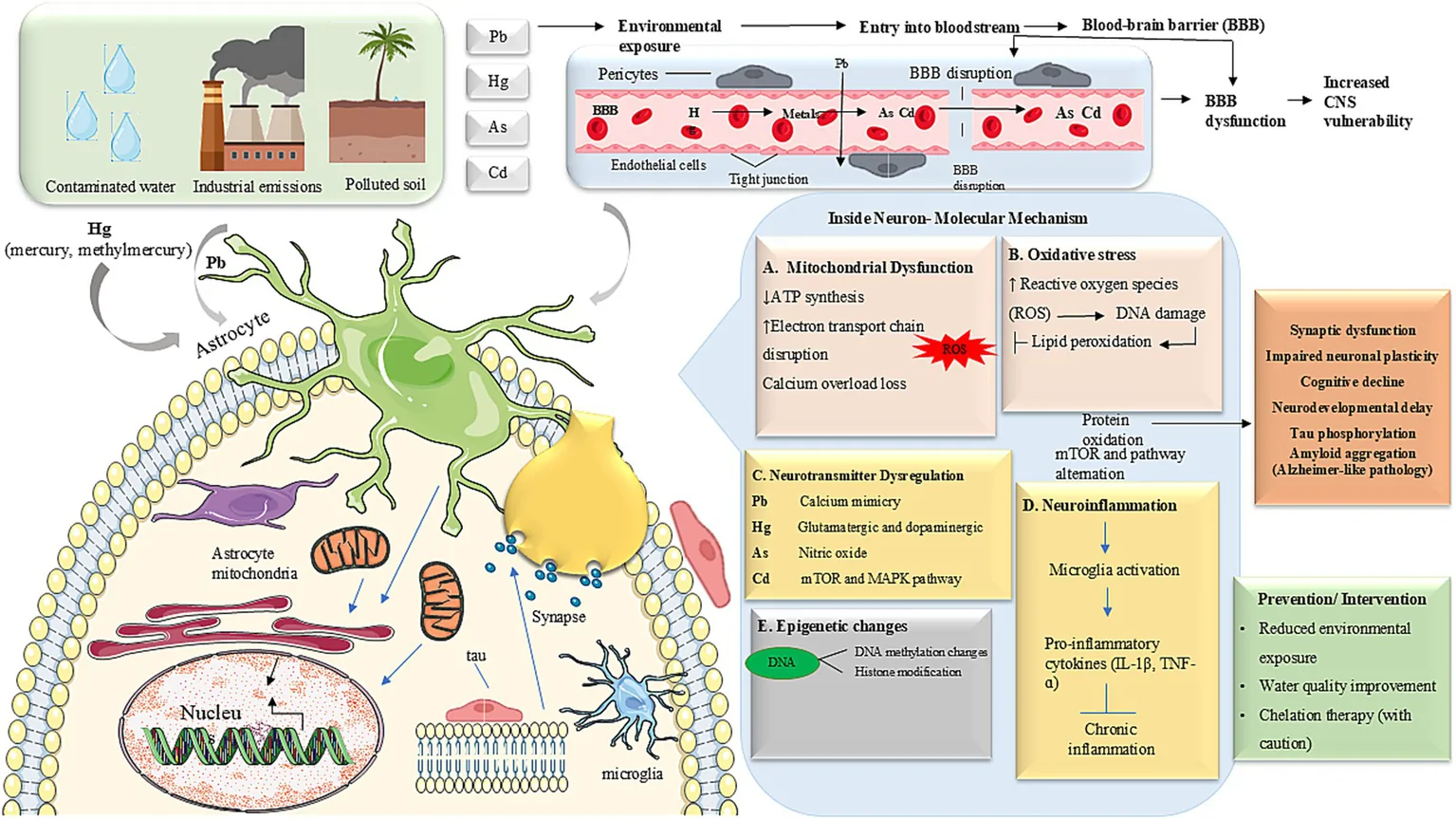
Environmental exposures across the lifespan linking neurodevelopmental and neurodegenerative brain disorders. Environmental contaminants such as lead (Pb), mercury (Hg), arsenic (As), and cadmium (Cd) originate from polluted water, industrial emissions, and contaminated soil. These metals enter systemic circulation and disrupt the blood–brain barrier (BBB) by damaging endothelial tight junctions, facilitating their accumulation in the central nervous system (CNS). Within the brain, heavy metals interact with neurons and glial cells, initiating multiple pathological pathways. They induce mitochondrial dysfunction by impairing the electron transport chain and ATP synthesis, leading to neuronal energy deficits. Concurrently, excessive generation of reactive oxygen species (ROS) causes oxidative stress, leading to lipid peroxidation, DNA damage, and protein oxidation. Heavy metals also disturb neurotransmitter signaling and activate microglia-mediated neuro-inflammation through cytokine release (e.g., IL-6, TNF-*α*). Additionally, epigenetic alterations such as DNA methylation and histone modification contribute to persistent dysregulation of neuronal gene expression.

Heavy metals such as lead (Pb), mercury (Hg), arsenic (As), and cadmium (Cd) remain a major source of environmental neurotoxins due to legacy pollution, contaminated water and soil, and industrial emissions (20). Environmental toxicants that accumulate in body tissues and cross protective biological barriers act as potent disruptors of synaptic integrity, neuronal homeostasis, and cognitive function, thereby contributing to neurodevelopmental impairments and neurodegenerative disease across the lifespan (21). Lead, one of the most extensively studied neuro-toxicants, especially in children, has been linked to reduced IQ, executive dysfunction, attention problems, and impaired synaptogenesis, all of which are associated with even low levels of exposure during critical stages of brain development. Lead can mimic and compete with calcium ions in neurotransmitter release and in calcium-dependent signaling, both of which are necessary for neural plasticity (22). Additionally, Pb induces inflammation, oxidative stress, and mitochondrial dysfunction, all of which affect neuronal viability. Mercury, especially in its organic form methylmercury, acts as a lipid-soluble neurotoxin that readily crosses the blood–brain barrier (23). After entering the central nervous system, mercury accumulates in neuronal membranes, inhibits mitochondrial ATP synthesis, generates reactive oxygen species (ROS), and interferes with neurotransmission, particularly glutamatergic and dopaminergic signaling. Arsenic, a metalloid commonly present in contaminated drinking water, disrupts cellular energy metabolism and induces oxidative stress (24). Its neurotoxic effects include disrupted synaptic function, impaired neuronal differentiation, and disrupted nitric oxide signaling—all processes vital to hippocampus-dependent learning and memory. Peripheral neuropathy, cognitive decline, and chronic long-term alterations in the maturation of neural circuits are associated with chronic exposure to arsenic (25). In addition, cadmium is known to exert strong neurotoxic effects. Exposure to Cd increases ROS production and depletes endogenous antioxidants, leading to oxidative damage to neuronal proteins, DNA, and lipids. Cd affects calcium homeostasis, alters neuronal signaling pathways such as mTOR and MAP kinase, and may lead to apoptosis (26). There is increasing evidence that exposure to cadmium increases the risk of age-related cognitive decline in adults and leads to cognitive delays in children. These heavy metals have overlapping pathways that converge on common targets of neurodegenerative diseases, including circulating toxins and inflammatory mediators; trigger neuro-inflammation through microglial activation and pro-inflammatory cytokine release; and promote epigenetic modifications that reprogram gene expression related to neuronal resilience and plasticity (27). Heavy metals have direct neurotoxic effects and induce disease mechanisms that interact in multiple molecular pathways. High levels of Pb, Hg, As and Cd promote the generation of ROS and disrupt the body’s antioxidant system, resulting in oxidative damage to lipids, proteins and nucleic acids. The disruption of ATP production, calcium homeostasis, and cellular bioenergetics resulting from oxidative injury makes neurons especially vulnerable to degeneration. Chronic neuro-inflammation is perpetuated by the release of pro-inflammatory cytokines and reactive mediators in response to heavy metal exposure, which stimulates microglia and astrocytes (28). Heavy metals, in turn, activate microglia and astrocytes, leading to the release of pro-inflammatory cytokines and reactive mediators that sustain chronic neuro-inflammation. Chronic oxidative stress and inflammation also disrupt other systems of protein quality control, such as the ubiquitin-proteasome system and the autophagy-lysosomal pathway, leading to the accumulation of misfolded proteins, such as amyloid-β, tau, and α-synuclein. These pathological processes compromise synapse function, induce apoptotic signaling pathways and lead to the progressive death of neurons in neurodegenerative diseases (29). Environmental toxicants induce neurotransmitter dysregulation, affecting the dopaminergic, glutamatergic, and cholinergic systems, disrupt the BBB, and increase CNS vulnerability (30). BBB dysfunction induced by heavy metals facilitates further heavy metal entry and inflammatory cell infiltration, which aggravates neurodegenerative cascades such as tau phosphorylation and amyloid aggregation, which are characteristic of AD (31). The key public health initiatives include limiting occupational exposure, improving water quality, and reducing environmental emissions. Chelating agents will help mobilize heavy metals at the individual level in cases of acute poisoning, but their use must be balanced against the risks of redistribution and possible depletion of essential metals (32). The major cellular mechanisms through which heavy metals induce neurotoxicity and contribute to neurodegenerative pathology are summarized in Figure 1. The major environmental exposures, their common sources, underlying cellular mechanisms, and associated neurological outcomes are summarized in Table 1.

**Table 1:** Environmental exposures and brain disorder mechanisms.

S. no.	Exposure	Common sources	Cellular mechanisms	Neurological outcomes	Type of evidence	References
1.	Air pollution (PM2.5, NOx, ozone)	Vehicle emissions, industry, biomass burning	Oxidative stress, neuroinflammation, BBB disruption, mitochondrial dysfunction	Cognitive decline, autism risk, Alzheimer’s disease	Epidemiological + experimental	(128)
2.	Lead	Paint, contaminated water, and soil	Synaptic dysfunction, calcium signaling disruption, and ROS production	Intellectual disability, behavioral disorders, neurodegeneration	Clinical + toxicological	(129)
3.	Mercury	Fish contamination, industrial waste	Protein misfolding, mitochondrial damage, neuronal apoptosis	Motor impairment, memory deficits	Human + animal studies	(130)
4.	Arsenic	Groundwater, pesticides	Epigenetic alterations, oxidative injury, and neuroinflammation	Developmental delay, cognitive impairment	Population + mechanistic	(131)
5.	Cadmium	Cigarette smoke, industrial exposure	Calcium imbalance, mitochondrial toxicity, lipid peroxidation	Neurodevelopmental deficits, Parkinsonian features	Experimental + observational	(132)
6.	Pesticides (e.g., paraquat, rotenone)	Agriculture, occupational exposure	α-synuclein aggregation, mitochondrial complex I inhibition	Parkinson’s disease	Case–control + animal models	(133)
7.	Industrial solvents	Paints, cleaning agents	Lipid membrane damage, neurotransmitter imbalance	Neurobehavioral dysfunction	Occupational cohort	(134)
8.	Endocrine disruptors	Plastics (BPA), cosmetics	Hormonal signaling disruption, epigenetic modulation	Neurodevelopmental disorders	*In vivo* + epidemiological	(135)

#### 3.1.1 Sources, exposure pathways, and global epidemiology of heavy metals

Heavy metals and metalloids are among the most common environmental contaminants worldwide and pose a public health threat due to their persistence, bioaccumulation, and neurotoxicity. While exposure has been mitigated in many developed nations, global industrialization, mining, agriculture, fossil fuel use, e-waste recycling and poor waste management are major sources of environmental contamination in many areas. There are several routes of human exposure to heavy metals. One of the most frequent ways of exposure is ingestion of contaminated drinking water, food products, and agricultural crops. The presence of arsenic in groundwater is a major issue in many parts of South Asia, and bio-magnification in aquatic food chains results in mercury contamination of commonly consumed fish and seafood products (33). Contaminated soil, old water distribution systems, industrial emissions, batteries, and electronic junk can all lead-to-Pb exposure. Tobacco smoke, industrial processes, phosphate fertilizers, and contaminated food are the main sources of cadmium exposure. Exposure to heavy metals in the air through inhalation of dust and in the workplace (e.g., in mines, smelting, battery making and metal-working) adds to the total exposure. Despite progress in environmental regulation, heavy metal exposure remains a significant global problem (34). Millions of people are still exposed to levels exceeding recommended safe limits, especially in low- and middle-income countries where environmental monitoring and remediation may be limited. Children, pregnant women and older adults are particularly susceptible because they are physiologically more susceptible and may have a greater lifetime risk of neurological sequelae. Chronic heavy metal exposure has been consistently associated with cognitive deficits, cognitive decline, decreased intelligence quotient (IQ), neurodevelopmental disorders, behavioral abnormalities and risk for age-related neurodegenerative diseases in epidemiological studies. There is a growing awareness of the neurological consequences of cumulative exposure over the lifetime, and efforts to prevent them, along with environmental surveillance and public health measures to limit exposure and its long-term neurotoxic effects (35).

#### 3.1.2 Role of astrocytes and microglia in heavy metal-mediated neurodegeneration

Astrocytes and microglia play key roles in maintaining CNS homeostasis and are central to the neurodegenerative processes triggered by heavy metal exposure (36). Heavy metals, including Pb, Hg, As and Cd, have a dramatic effect on glial cell function, causing chronic neuro-inflammatory responses that can lead to a gradual progression of neuronal damage. Resident immune cells of the CNS, microglia, are highly responsive to environmental toxicants and rapidly change their phenotype to a pro-inflammatory state in response to heavy metal exposure. When activated, microglia produce high levels of inflammatory mediators, including TNF-α, IL-1β, IL-6, nitric oxide, and ROS, thereby exacerbating local inflammation and promoting neuronal dysfunction (37). An ongoing, chronic pattern of microglial activation exacerbates oxidative stress and leads to further damage to synapses, resulting in a vicious cycle of neuro-inflammation and neurodegeneration. Astrocytes are also involved in mediating heavy metal toxicity in the nervous system. Under physiological conditions, astrocytes play roles in maintaining neuronal survival, recycling neurotransmitters, maintaining ion homeostasis, providing metabolic substrates, and regulating BBB integrity. But exposure to high levels of the metals disrupts these protective functions and triggers a hyper-responsive astrogliosis. Reactive astrocytes show a depletion of antioxidants, enhanced secretion of pro-inflammatory cytokines and chemokines, and changes in glutamate uptake. Glutamate uptake by astrocytes is impaired, increasing neuronal calcium influx and activating cell death pathways, thereby causing excitotoxicity. Heavy metal-induced astrocytic dysfunction also alters BBB stability, allowing circulating inflammatory mediators to enter the CNS and further exacerbate inflammation (38). There is a bi-directional relationship between activated microglia and reactive astrocytes, and this relationship is known to play a major role in disease progression. Neurotoxic microglial phenotypes can be triggered by cytokines released from activated microglia, while microglia can be further activated by inflammatory mediators from astrocytes. This bi-directional signaling further increases oxidative stress, mitochondrial dysfunction and neuronal damage. Furthermore, chronic glial activation disrupts protein clearance mechanisms, such as autophagy and lysosomal degradation, leading to pathogenic protein aggregation, including amyloid-β, hyper-phosphorylated tau, and α-synuclein. By regulating these interrelated processes, astrocytes and microglia play key roles in linking heavy metal exposure to chronic neuro-inflammation, neuronal death, and neuro-degenerative diseases (28).

#### 3.1.3 Heavy metal-induced epigenetic modification and neurological dysfunction

Heavy metals have long-term effects on neural development, neuronal function, and disease susceptibility, mediated by epigenetic dysregulation. Epigenetic changes are alterations in gene expression that do not change the DNA sequence and can involve DNA methylation, histone modifications, chromatin remodeling, and non-coding RNA-mediated regulation (39). The growing body of evidence for epigenetic changes caused by environmental neuro-toxicants such as Pb, Hg, As, and Cd, and their association with both neurodevelopmental abnormalities and neurodegenerative processes. Heavy metals disrupt DNA methyltransferase activity and the availability of methyl donors, thereby interfering with DNA methylation patterns (40). Exposure to Pb, As, and Cd has been reported to lead to aberrant methylation of genes involved in synaptic plasticity, neuronal differentiation, oxidative stress responses and neuro-immune regulation (41). These changes can affect normal brain development during important developmental windows, making them more vulnerable to cognitive dysfunction later in life. Heavy metals can also alter histone acetylation and methylation, thereby affecting chromatin accessibility and transcription. These alterations involve genes that play roles in neuronal survival, mitochondrial function, inflammatory signaling, and the cellular stress response. Another important epigenetic mechanism altered by heavy metal exposure involves microRNAs (miRNAs) and other non-coding RNAs (42). Dysregulation of miRNA expression has been linked to changes in neuronal maturation, synaptic communication, and neuro-inflammatory responses. These epigenetic changes can last a lifetime, which could be the reason why neurotoxic effects occur many years after exposure in environmentally related diseases. It is important to note that epigenetic changes could act as a mechanism between environmental exposures and oxidative stress, mitochondrial dysfunction, neuro-inflammation and protein aggregation. Chronic dysregulation of genes that underlie antioxidant defenses, autophagy and immune homoeostasis can increase the vulnerability of neurons and contribute to the accumulation of pathological proteins, like amyloid-β, tau, and α-synuclein. Epigenetic changes are also potentially reversible and therefore represent targets for therapeutic intervention and biomarker generation (43). Epigenomic studies will be crucial in the future to understand the role of epigenomics in neurological deficits throughout life, which can result from exposure to heavy metals.

### 3.2 Air pollution and particulate matter

**Figure 2:**
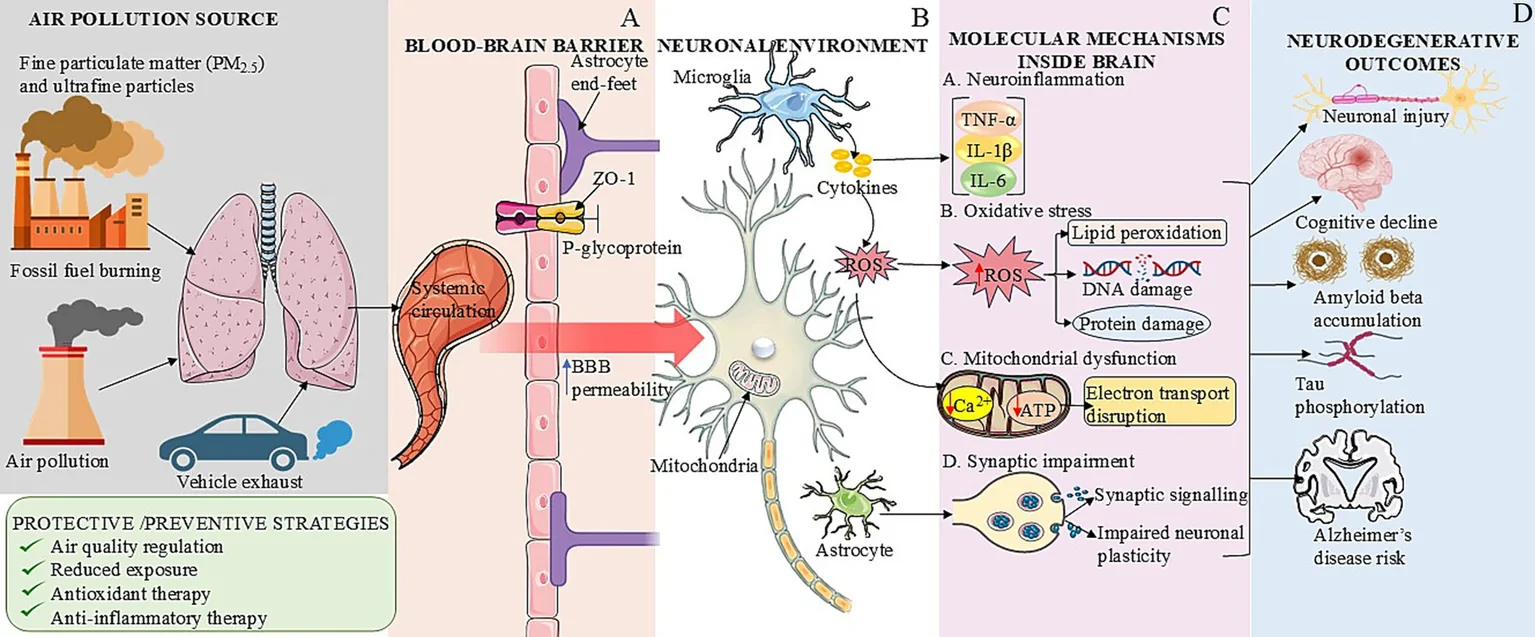
Schematic illustration of the impact of air pollution–derived fine particulate matter and ultrafine particles on brain health. Inhaled pollutants from fossil fuel combustion and vehicle exhaust enter systemic circulation and disrupt the blood–brain barrier (BBB), increasing permeability. This facilitates the entry of toxic metals and particles into the central nervous system (CNS). Within the neuronal environment, activated microglia and astrocytes release pro-inflammatory cytokines (TNF-α, IL-1β, IL-6) and generate reactive oxygen species (ROS), triggering neuro-inflammation and oxidative stress. These processes cause lipid peroxidation, DNA and protein damage, mitochondrial dysfunction, ATP depletion, and Ca^2+^ imbalance, thereby impairing synaptic signaling and neuronal plasticity. Ultimately, these molecular events contribute to amyloid-β accumulation, tau phosphorylation, neuronal injury, cognitive decline, and increased Alzheimer’s disease risk.

Air pollution, in particular, fine particulate matter (PM2.5) and ultrafine particles, has become a ubiquitous environmental risk factor with negative impacts on brain health and is implicated in the etiology of neurodegenerative and neurodevelopmental disorders. Long-term exposure to high levels of PM2.5 is associated with accelerated cognitive decline and a high incidence of dementia in old age, including Lewy body dementia and AD, according to epidemiological evidence (44). Particles less than 2.5 microns in diameter are known as fine particulate matter and are largely formed through industrial processes, vehicle emissions, and the burning of fossil fuels. Such particles can reach the systemic circulation and penetrate deep into the respiratory tract due to their small size, thereby providing a route to the central nervous system (45). Chronic exposure to PM2.5 has been associated with structural brain changes and reduced cognitive function, suggesting widespread effects on brain integrity. Neuro-inflammation and disruption of the blood–brain barrier are two interrelated mechanisms implicated in PM2.5-induced neurotoxicity at the cellular level. The BBB maintains neuronal homeostasis by strictly controlling the passage of substances into the brain under physiological conditions (46). PM2.5 exposure increases BBB permeability by compromising tight junction proteins, such as zonula occludens-1 (ZO-1), and reducing efflux transporters, such as P-glycoprotein, thereby facilitating the entry of inflammatory mediators and ultrafine particles into the CNS, as demonstrated in Figure 2A (47). Peripheral inflammatory mediators, ultra-particles, and other toxins are more readily able to cross the brain parenchyma because of this impaired barrier function. The activation of innate immune cells, particularly microglia, by particulate matter in the central nervous system environment leads to an extended pro-inflammatory response characterized by increased cytokine levels, including interleukin-1*β*, interleukin-6, and tumor necrosis factor *α*, as illustrated in Figure 2B (48). As shown in Figure 2C, PM2.5-induced stress and mitochondrial dysfunction impair neuronal bio-energetic and synaptic integrity, thereby contributing to progressive neuronal injury. There is also evidence linking pollution-associated inflammation to hallmark neurodegenerative pathology: chronic PM2.5 exposure has been associated with increased accumulation of pathogenic proteins, including phosphorylated tau and amyloid-β, thereby linking environmental pollution to hallmark neurodegenerative pathology, as shown in Figure 2D (49). The most direct way to reduce the neurotoxic effects of air pollution is to limit exposure through air quality regulation and public health policies. Furthermore, anti-inflammatory and antioxidant treatments may, in part, be responsible for reducing PM-induced neuro-inflammation and BBB disruption in experimental models, suggesting a potential supplementary strategy for at-risk populations (50). Future mechanistic and clinical studies are needed to elucidate specific inflammatory pathways and to develop precision therapeutic strategies to protect the integrity of the nervous system under environmental stress (51). The mechanisms by which air pollution-derived particulate matter disrupts the BBB and induces neuro-inflammation, oxidative stress, mitochondrial dysfunction, and synaptic impairment, leading to neurodegeneration, are illustrated in Figure 2.

## 4 Shared molecular and cellular mechanisms linking environment to brain pathology

Proteostasis (protein homeostasis) is the cellular mechanism that supports the synthesis, folding, trafficking, interaction, and degradation of proteins to ensure proper protein quality control and cellular function (52). This network encompasses molecular chaperones, which help proteins fold correctly, the ubiquitin-proteasome system, which degrades short-lived or misfolded proteins, and the autophagy-lysosomal pathway, which degrades damaged proteins and organelles (53). These processes are impaired when a protein becomes misfolded, its transport is disrupted, it interacts abnormally with other proteins, it is not cleared, or it aggregates to form a toxic protein, thereby causing proteostasis dysfunction. Multiple components of the proteostasis network can be dysregulated by environmental stressors, including heavy metals, pesticides, and air pollution, thereby contributing to neuronal dysfunction and neurodegeneration (54).

### 4.1 Oxidative stress and mitochondrial dysfunction

The two major, interrelated processes by which environmental exposures lead to neuronal and glial pathology, in both neurodevelopmental and neurodegenerative disorders, are oxidative stress and mitochondrial dysfunction. The brain is particularly susceptible to redox imbalance due to its high metabolic demands and relatively weak antioxidant defenses, especially when exposed to environmental stressors over time, including industrial chemicals, heavy metals, and air pollution (55). Disruption of redox homeostasis in neurons and glial cells leads to excessive generation of reactive oxygen species. This directly affects cellular bioenergetics and initiates a cascade of pathological processes that impair brain function. ROS that includes hydrogen peroxide (H_2_O_2_), superoxide anions (O^2−^), and hydroxyl radicals (OH) are naturally occurring byproducts of mitochondrial oxidative phosphorylation (56). Environmental toxicants disrupt the electron transport chain (ETC), leading to an abnormal increase in reactive oxygen species. ETC complexes I–IV produce ATP by facilitating electron transfer to oxygen under physiological conditions (57). Metal ions and pesticides are examples of environmental stressors that can block electron flow, leading to increased electron leakage and subsequent reduction of oxygen to ROS. Excessive ROS accumulation overwhelms endogenous antioxidant systems, such as glutathione and superoxide dismutase, leading to oxidative modification of lipids, proteins, and nucleic acids in neurons and glial cells (58). Overproduction of ROS actively alters mitochondrial structure and function, leading to bioenergetic failure rather than a passive consequence of metabolic dysregulation. The primary source of energy, ATP production, for neuronal signaling, neurotransmitter cycling, and ion homeostasis, is impaired by excessive ROS, which also causes damage to critical components of the ETC and mitochondrial DNA (mtDNA) (59). Apoptotic signaling and glial dysfunction result from reduced mitochondrial membrane potential, improper opening of permeability transition pores, and decreased calcium buffering capacity. Since neurons rely on oxidative phosphorylation and have a high ATP demand, failure of bioenergetics has serious repercussions (60). The substantia nigra dopaminergic neurons, which are especially vulnerable in PD, have a high baseline production of ROS and are particularly susceptible to oxidative stress from environmental pollutants. In these neurons, mitochondrial damage mediated by ROS is responsible for axonal degeneration, impaired synapses, and ultimately cell loss—all features of neurodegenerative pathology (61). Oxidative stress contributes to worsening pathology associated with tau and amyloid-β, inhibits mitochondrial respiration, and contributes to feedback loops of degeneration in AD (62). ROS dysfunction also occurs in glial cells, including microglia and astrocytes. Prolonged oxidative stress drives microglia toward a pro-inflammatory phenotype, increasing ROS production and promoting cytokine-mediated neurotoxicity, while compromising astrocytic support of neuronal metabolism and glutamate clearance (63). This glial dysfunction accelerates the deterioration of neural structure and function through a vicious cycle that reinforces oxidative stress and inflammation. Crucially, the relationship between oxidative stress and mitochondrial dysfunction is dynamic; once the process begins, elevated ROS further destabilize mitochondria, leading to more ROS and gradually worsening cell bioenergetics. This self-propagating loop drives many neuro-inflammatory and neurodegenerative processes, and, as a consequence, these processes are attractive targets for treatment (64).

### 4.2 Neuro-inflammation and immune dysregulation

Environmental risk factors are implicated in both neurodevelopmental alterations and neurodegenerative diseases through neuro-inflammation. The innate immune cells resident in the CNS is called microglia and lie at the heart of the neural immune response (65). Microglia sense the microenvironment, clear cellular debris, and support synaptic refinement when homeostasis is maintained. However, a state of microglial priming, in which microglia are hypersensitive to subsequent stimuli, can be induced by repeated or prolonged environmental insults, such as air pollution, toxicants, or metabolic stress (66). The pro-inflammatory phenotype of primed microglia is characterized by increased expression of reactive mediators and cytokines that sustain neuro-inflammation over longer periods and disrupt neuronal circuitry. Priming alters the threshold and intensity of microglial responses, leading to an overreaction to even small secondary stimuli (67). Long-term low-grade inflammation and previous exposures to the environment sensitize microglia and lead to chronic inflammatory cascades promoting accelerated neurodegenerative pathology. This is a mechanistic feature associated with age-related disorders. Peripheral-central immune crosstalk is necessary to sustain neuro-inflammation, in addition to CNS-resident responses (68). Infiltration of peripheral immune cells, such as T lymphocytes and monocytes, into the brain parenchyma is facilitated by disruption of the blood–brain barrier, which is itself induced by oxidative stress or exposure to toxicants. Once inside the central nervous system, these cells interact with astrocytes and microglia, leading to increased local inflammatory signaling, synaptic dysfunction, and neuronal death (69). Peripheral cytokines and chemokines may also influence central immunological responses without infiltrating the body. Circulating inflammatory mediators can affect transport mechanisms across the BBB or signal to the BBB via endothelial cells. Peripheral immune responses are induced by the release of damage-associated molecular patterns from CNS injury or neurodegenerative processes, whereas systemic inflammation can induce CNS inflammation by signaling via cytokines and by modulating the BBB (70). The progression of various neurologic pathologies is driven by a self-propagating cycle of immune dysregulation induced by this feedback loop. Microglial phenotypes are not all or nothing—they are neuroprotective to neurotoxic states depending on the peripheral signals and the environment. Therapeutic approaches to reduce chronic neuro-inflammation in neurodegenerative diseases that target peripheral immune contributions, maintain the integrity of the blood–brain barrier, or modify microglial activation are increasingly being studied (71).

## 5 Environmental drivers of neurodegenerative diseases

### 5.1 Alzheimer’s disease

**Figure 3:**
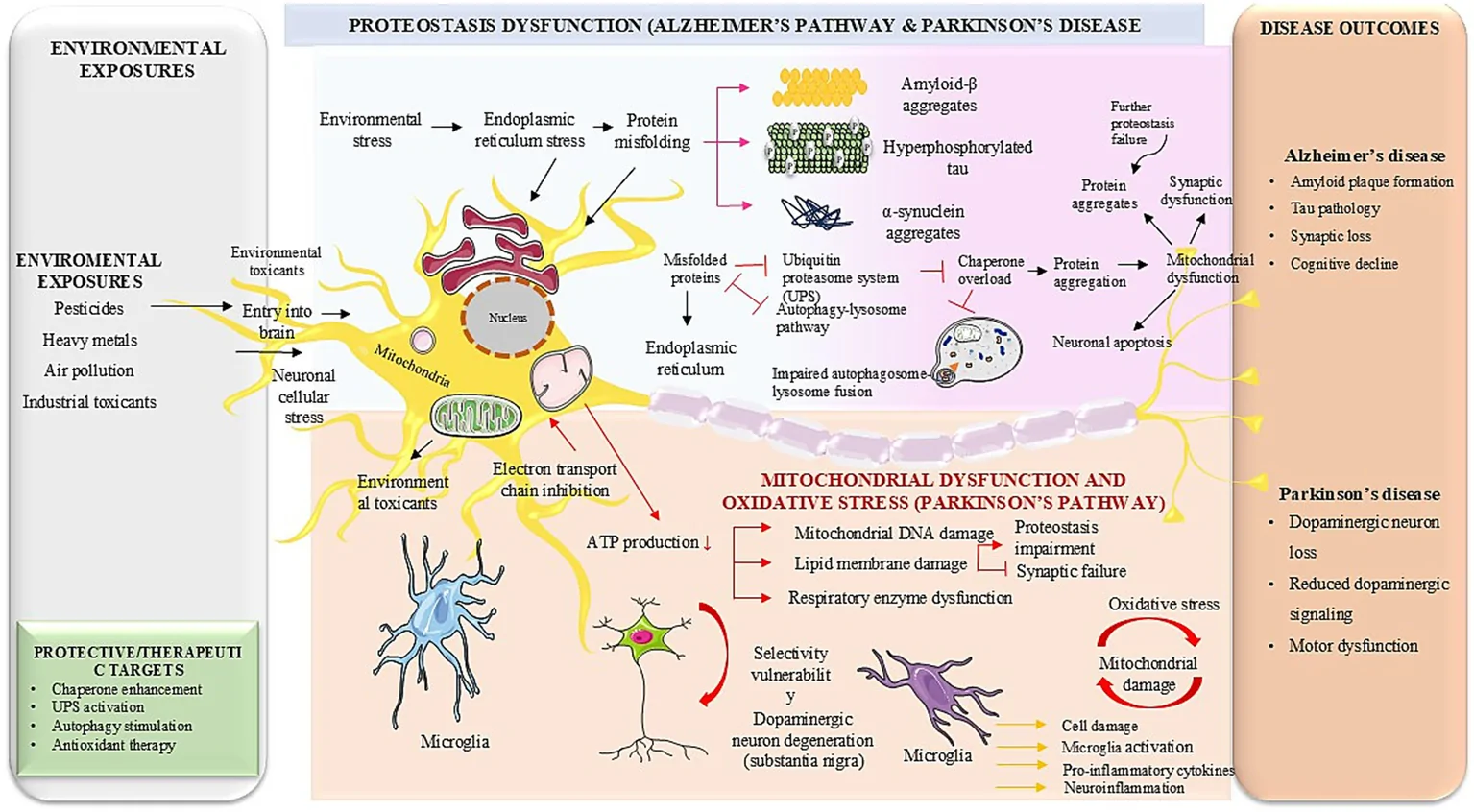
Schematic representation of environmental exposure-induced proteostasis dysfunction contributing to neurodegenerative disorders. Environmental toxicants such as pesticides, heavy metals, air pollutants, and industrial chemicals enter the brain and trigger neuronal cellular stress, including endoplasmic reticulum stress and mitochondrial dysfunction. These stressors impair protein quality-control systems, including the ubiquitin-proteasome system and the autophagy-lysosome pathway, leading to the accumulation of misfolded proteins. Consequently, pathogenic aggregates such as amyloid-β, hyper-phosphorylated tau, and α-synuclein accumulate, promoting protein aggregation, synaptic dysfunction, and neuronal apoptosis. Mitochondrial DNA damage, reduced ATP production, lipid membrane damage, and oxidative stress further exacerbate neuronal injury. These molecular alterations ultimately result in neuro-inflammation, dopaminergic neuron degeneration, impaired dopaminergic signaling, cognitive decline, and increased risk of Alzheimer’s disease and Parkinson’s disease.

The abnormal folding of proteins is a hallmark of major neurodegenerative diseases, including AD, and results in disruption of the cell’s protein quality control (proteostasis) network, which controls protein synthesis, folding, transport to the correct location, protein–protein interactions and degradation (72). These processes are disrupted, leading to misfolded proteins and toxic aggregates that build up, causing neuronal dysfunction (73). Amyloid-β, hyper-phosphorylated tau, and α-synuclein are examples of characteristic aggregates (74). In pathological situations, this balance is disrupted, leading to the formation of toxic oligomers, insoluble fibrils, and neuronal dysfunction (75). Normally, these proteins fold into precise three-dimensional structures required for function. Environmental factors that can disrupt protein folding and degradation, such as pesticides, heavy metals, and air pollutants, can accelerate proteostasis breakdown. The endoplasmic reticulum dysfunction, oxidative damage, and cellular stress induced by these stressors overwhelm the chaperone systems responsible for proper folding and the maintenance of quality (76). Aggregation-prone proteins continue to aggregate due to reduced proteasomal and autophagic system efficiency, and to chaperone sequestration by misfolded substrates. The UPS uses polyubiquitination and the 26S proteasome to degrade short-lived, soluble, misfolded proteins in normal conditions (77). Aggregates that are insoluble and long-lived are engulfed by autophagy, particularly macro-autophagy, and are then delivered to lysosomes for destruction. When these pathways are disrupted by environmental insults, clearance of tau, α-synuclein, and β is reduced (78). This results in prolonged protein aggregation, mitochondrial dysfunction, and synapse dysfunction, ultimately leading to neuronal apoptosis. Protein aggregates may also contribute to autophagy dysfunction; pathological tau and α-synuclein interfere with lysosomal and autophagosome fusion, leading to a self-reinforcing cycle of proteostasis dysfunction (79). This cycle not only favors the accumulation of harmful protein species but also renders neurons more susceptible to other forms of stress, such as neuro-inflammation and oxidative damage, which are often exacerbated by environmental factors. The pathological interaction between the clearance systems and misfolded proteins identifies important molecular targets for treatment. In preclinical models of the disease, enhancing proteostasis by pharmacologically modulating chaperone expression, activating UPS components, or stimulating autophagy has shown promise (80). Dietary polyphenols and small molecules that enhance autophagic flux or proteasomal activity have been associated with increased degradation of α-synuclein and related aggregates, suggesting methods to offset environmental factors that contribute to neurodegenerative processes. Environmental toxicants can disrupt proteostasis and promote amyloid-β aggregation, tau hyperphosphorylation, mitochondrial dysfunction, and oxidative stress, which collectively contribute to AD, as shown in Figure 3.

### 5.2 Parkinson’s disease

PD is a progressive neurodegenerative disorder, the main symptom of which is destruction of dopaminergic neurons of the substantia nigra pars compacta to the striatal level of dopamine, motor control, and non-motor dysfunction affecting a broad range of functions, which are manifestations of the inability of integrated circuitry in the basal ganglia (81). Intrinsic aging and genetic susceptibility establish a biological predisposition to disease; environmental toxicants are powerful external modulators that accelerate neuronal damage and determine the course of the disease. A long-term exposure to agricultural chemicals has been steadily linked to an increased risk of Parkinson’s disease, and these findings depict harmful environmental exposures that trigger pathogenic cascades well before clinical manifestations are observed (82). Numerous pesticide molecules accumulate at mitochondrial dysfunction, which is a key pathological agent of dopaminergic degeneration, since the survival of neurons relies on the effectiveness of oxidative phosphorylation and the continuity of ATP production in mitochondria (83). Some of the toxicants block electron transport chain pathways, thus affecting the flow of electrons, and decreasing energy production, which in turn increases excessive production of reactive oxygen species, leading to damage of mitochondrial DNA, membrane lipids, and respiratory enzymes, which destabilize bioenergetics of the cell (84). Such a defect in mitochondrial activity is especially harmful to dopaminergic neurons, since the metabolism of dopamine is already associated with the production of reactive intermediates; that is, these cells are already operating under endogenously elevated oxidative stress, which makes them less resistant to further toxic damage. Mitochondrial impairment is exacerbated by proteostatic alterations, as mitochondrial oxidative damage disrupts the protein-folding apparatus and overloads degradation pathways that eliminate misfolded or aggregated proteins (85). Inefficiency of clearance pathways, aggregation-prone protein buildup, impaired vesicular trafficking, and disrupted neuronal communication reduce neuronal communication and accelerate structural degeneration. These disruptions within cells further stimulate microglial cells, which respond to cellular injury by releasing pro-inflammatory cytokines and reactive mediators to amplify oxidative stress and extend neuro-inflammation, thereby forming a vicious cycle of cell death (86). Notably, individuals differ in their vulnerability to toxin-related neurodegeneration due to genetic differences in detoxification capacity, mitochondrial quality control, and antioxidant defenses, which determine neurons’ ability to overcome environmental stressors. The absence of these protective mechanisms leads to progressive loss of neuron resilience to cumulative toxic exposure, resulting in axonal dysfunction, synaptic failure, and eventual dopaminergic cell death (87). This combined model demonstrates that pesticides and mitochondrial toxins do not function through discrete mechanisms but rather, they result in interdependent molecular pathways of oxidative imbalance, energy collapse, proteostasis perturbation and immune activation that led to the development and progression of PD, and make environmental modulation an important determinant of the development and course of the disease (88). Exposure to environmental toxicants may induce mitochondrial damage, oxidative stress, and *α*-synuclein aggregation, leading to dopaminergic neuronal degeneration and PD progression, as shown in Figure 3.

## 6 The gut-brain axis as an environmental interface

The gut-brain axis (GBA) is a well-organized two-way communication system that connects gastrointestinal physiology and the function of the central nervous system in a highly coordinated system of neural, immunological, endocrine and metabolic signals, thereby creating a significant biological interface of exposure to the environment, on which the brain health depends during the life span (89). This system comprises intestinal microbiota, the enteric nervous system, vagal pathways, circulating metabolites, immune mediators, and neuroendocrine regulators, all working in symbiotic harmony to maintain physiological equilibrium and neural stability (90). Microorganisms living in the gut are quick to react to external stimuli. They can thus be considered environmental biosensors that convert dietary patterns, pollutant exposure, lifestyle practices, and chemical toxicants into molecular signals that regulate neuronal activity, synaptic plasticity, and neuro-immune homeostasis (91). The interactions between microbiomes and the environment are of particular concern during early development, when colonization of the infant’s intestinal tract influences immune development, metabolic programming, and neural circuitry (92). The temporal dynamics of gut microbiota changes are also crucial to the neurological outcome throughout the lifespan. Dysbiosis in early life has the potential to disrupt immune maturation, synapse development and neurobehavioral programming and consequently to predispose to neurodevelopmental disorders like ASD and ADHD. Conversely, a chronic imbalance in the microbiome in adulthood and aging can be progressively associated with systemic inflammation, oxidative stress, reduction in metabolites and pathways of protein aggregation that are linked to AD and PD. From these observations, it is clear that neurological vulnerability related to the microbiome is a dynamic process that may vary depending on the developmental stage at which the environment is disrupted. Material stress, antibiotic exposure, dietary imbalance, or toxin ingestion can alter microbial diversity and metabolite profiles, thereby affecting signaling pathways that control neurodevelopment (93). These disruptions affect neurotransmitter synthesis, the myelination and pruning of synapses, and, hence, cognitive maturation and behavioral control. Microbial products that reach the systemic circulation can overcome physiological barriers and alter neuronal gene expression and epigenetic mechanisms associated with long-term neurobiological vulnerability (94). Dietary components and xenobiotic chemicals also influence this interface by altering the composition and metabolic activity of microbes, which, in turn, influence the nervous system’s physiology through a cascade of interacting biochemical reactions. Intestinal microbes process the nutrient substrates into short-chain fatty acids (SCFAs), vitamins, and neuro-modulatory molecules that regulate inflammation, integrity of the mitochondria, and the blood–brain barrier, and this is a good demonstration of the interaction between nutritional environments and the activity of the brain (95). Microbial enzymatic pathways may also be altered by exposure to environmental toxicants and synthetic chemicals, leading to increased pro-inflammatory metabolites, reduced production of neuroprotective compounds, and impaired host-microbe symbiosis, thereby promoting systemic immune stimulation. Increased intestinal permeability facilitates the translocation of endotoxins into the circulation, thereby triggering peripheral immune responses that promote central inflammation and induce neuronal stress (96). The GBA disorder is important in both neurodevelopmental and neurodegenerative diseases, as chronic microbial imbalance perpetuates oxidative stress, immune activation, and metabolic disruptions that are common pathological processes in neurological diseases (97). Disrupted microbial signaling during development impairs synaptic organization and neural connectivity, thereby increasing the risk of developmental disorders. At the same time, chronic dysbiosis in adulthood enhances neuro-inflammatory cascades and protein aggregation pathways that drive age-related neurodegeneration (98). It is the accumulation of environmental exposures over time, integrated by gut-mediated signaling pathways, that constitutes physiological memory systems that determine disease vulnerability. According to this integrative model, the GBA is a key mediator between the outside world and the inside neurobiological response and thus, microbiome-directed interventions, dietary optimization, and decreasing the toxic exposures can be described as the promising options of maintaining neural resilience in different ages and stages of development (99).

## 7 Translational and clinical implications

Environmental neuroscience translational integration in clinical practice presents a basic neuroscience paradigm that can transform mechanistic understanding into action strategies to improve early diagnosis and predict and prevent neurological conditions across the life span. Neural integrity is affected by environmental factors through interconnected mechanisms of oxidative stress, mitochondrial dysfunction, immune activation, and proteostatic imbalance, and the recognition of these convergent mechanisms has enabled the identification of measurable biological indicators of exposure burden and early pathological change (100). The environmental biomarkers can therefore be considered useful as an indicator of the subclinical neural damage before manifestation of acute symptoms because, on the molecular level in the blood, cerebral spinal fluid, metabolic signature, or epigenomic alteration could be taken as a cumulative toxic exposure, inflammatory response, and the start of neuronal stress. Some markers suggested as early indicators of environmentally induced neuronal injury following exposure to heavy metals and neuro-inflammation include elevated blood lead levels, altered inflammatory cytokine levels, and oxidative stress markers, such as malondialdehyde and reduced glutathione. Measurement of these enables clinicians to monitor biological responses to environmental exposures, disease predisposition, and disease progression, thereby bridging the gap between experimental evidence and clinical evaluation (101). Imaging biomarkers have been used in populations exposed to PM2.5 and have shown reduced cortical thickness, white matter abnormalities, and accelerated cognitive decline, providing evidence supporting their use for early risk stratification. The idea of risk-stratification strategies extends this translational approach by co-aggregating environmental exposure history with genetic vulnerability, lifestyle, and molecular biomarker data to create individual risk profiles that better predict neurological vulnerability. Gut microbial metabolites like short-chain fatty acids and inflammatory markers associated with LPS are emerging as markers and targets for treating ASD and AD. There are significant variations in detoxification capacity, mitochondrial resilience, immune responsiveness, and epigenetic responsiveness among individuals, and these biological variations determine the extent to which environmental stressors affect the development and progression of disease (102). The combination of exposure evaluation with systems-level biological measurements enables the identification of high-risk groups before clinical effects appear and allows the avoidance of severe disease onset through specific interventions, e.g., reducing exposure, optimizing nutrition, applying antioxidants, or regulating inflammation. These precision-oriented frameworks make clinical practice more proactive, relying on prevention rather than reactive treatment, thereby helping minimize disease burden in the long term and enhancing quality of life (103). Public health and regulatory issues can be considered a critical extension of translational neuroscience, since environmental factors related to brain diseases often act at the population level, requiring collective policy action to restrict exposure to neurotoxins. The quality of air, industrial output, water safety, pesticide use, and occupational risks are all regulated standards that directly affect neurological health outcomes by minimizing cumulative toxic burden within a population (104). In support of applying preventive policies to reduce exposure to environmental contaminants during vulnerable developmental periods and aging, epidemiological evidence indicates associations between environmental contaminants and neurological morbidity. Assessment of scientific evidence on environmental regulations enhances risk assessment and informative guidelines aimed at protecting people at risk, such as pregnant women, children, and the elderly (105). The combination of clinical translation, personalized risk modeling, and evidence-based environmental regulation constitutes a coherent approach to reducing environmentally mediated neurological disease, as each component can intervene at different levels, from molecular detection to societal prevention. The proposed multidimensional approach highlights that the management of neurodevelopmental and neurodegenerative diseases cannot be effectively done without being innovative in its approach to therapeutic work and systematically addressing the risk factors in the environment, thus highlighting prevention as one of the main concerns of the future of neurological health care (106). Finding sensitive and reliable biomarkers is a key step to detecting environmentally induced neurological disorders at an early stage. Usually, clinical diagnosis is only possible after considerable neuronal damage has occurred, making it of great interest to develop molecular biomarkers that detect pathological changes before they become clinically apparent. Circulating inflammatory markers, oxidative stress markers, mitochondrial dysfunction markers, epigenetic markers, neurofilaments, and metabolite markers from environmental exposures are candidate markers. The gut-brain axis has also been shown to alter the gut metabolites, such as short-chain fatty acids, bile acids, and lipopolysaccharide (LPS)-associated inflammatory markers, which are also being investigated as potential markers of gut-brain axis dysfunction (74). Molecular methods are complemented by advances in neuroimaging techniques for mapping structural and functional changes in the brain that occur in response to environmental toxicant exposure before clinical symptoms. The ability to combine these biomarker platforms may enhance disease monitoring, patient stratification, and personalized risk assessment, especially for populations living with chronic environmental stressors.

## 8 Therapeutic and preventive perspectives

Therapeutic and preventive interventions for site-sensitive degenerative injuries of the central nervous system are increasingly focusing on mechanistic treatments at multiple biological levels, incorporating lifestyle changes, environmental risk factors, and molecularly guided therapies to maintain neuronal resilience and slow disease progression. Environmental exposures disrupt the brain’s homeostasis through integrated mechanisms of oxidative stress, mitochondrial dysfunction, immune response, and epigenomic remodeling, and the recognition of these commonalities has shifted treatment models from symptom control to preventive therapies (107). The reduced cumulative toxic burden, leading to a reduced response to neurodegenerative cascades, is achieved through interventions that reduce cumulative exposure to neurotoxic pollutants, industrial chemicals, and dietary contaminants. Lifestyle factors, such as physical activity, sleep regulation, stress reduction, and cognitive engagement, enhance endogenous neuroprotective systems by increasing mitochondrial efficiency, strengthening antioxidant defense, and promoting synaptic plasticity to overcome environmentally triggered cellular stress (108). Microbiome regulation and nutritional interventions are also preventive, as dietary components directly influence microbial structure and metabolic signaling, which in turn affect neuro-immune and neuroendocrine circuits. Antioxidant-, polyphenol-, omega-3 fatty acid-, and micronutrient-rich diets help sustain mitochondrial respiration and neuronal membrane stability, inhibit inflammatory signaling, and stimulate the formation of Mito-protective metabolites within microbial communities (109). Conversely, diets rich in processed foods, environmental pollutants, or xenobiotic remnants can promote dysbiosis, systemic inflammation, and metabolic dysregulation, thereby increasing neural susceptibility. Prebiotics, probiotics, and dietary interventions can be used to manipulate the gut microbiota to restore microbial balance, improve intestinal barrier integrity, and regulate metabolite production, which, in combination with neurochemical signaling, provide neuronal networks with resistance to environmental stress (110). Such microbiome-directed interventions have demonstrated potential to contribute to intestinal barrier integrity, modulate inflammatory signaling, and enhance neurochemical homeostasis in neurodevelopmental and neurodegenerative disorders. The identification of fundamental molecular pathways underlying environmental neurotoxicity has opened encouraging therapeutic avenues to alter the disease’s biological mechanisms. Anti-inflammatory interventions, which modulate neuro-immune signaling during microglial activation, can restrain chronic but regulated neuronal injury, whereas antioxidant interventions can dampen reactive oxygen species accumulation and maintain mitochondrial viability (111). Anti-inflammatory drugs or cytokine-targeting strategies could serve as a therapeutic modulation of microglial activation, helping reduce chronic neuro-inflammation caused by PM2.5 and other environmental toxicants. Antioxidants such as N-acetylcysteine, coenzyme Q10, and polyphenolic compounds have been shown to exert neuroprotective effects in experimental models, decreasing ROS accumulation and maintaining mitochondrial function. Environments that cause changes in DNA methylation or histone modification, which can be reversed by interventions that stabilize epigenetic regulation, can be countered. Interventions to improve mitochondrial quality control, increase biogenesis, or enhance electron transport chain efficiency can also be used to support cellular bioenergetics and inhibit apoptosis induced by environmental toxins (112). At the population level, reducing occupational exposure to pesticides and improving urban air quality may be relevant preventive measures to reduce the burden of Parkinson’s disease, dementia, and environmentally associated neurological dysfunction. A holistic approach to addressing environmental exposure, microbial balance, metabolic status, and intracellular signaling pathways is the most comprehensive therapy for supporting brain health over a lifetime, as the pathologic effects of neurological disorders result from the complex interplay between environmental stressors and underlying genetic susceptibility. These multidimensional approaches emphasize that prevention and therapy are not conceptually independent but mechanistically related, and that integrating environmental health policies and personalized biological treatments is essential for establishing long-term neuroprotection and neurological resilience (113).

### 8.1 Translational and preventive strategies for heavy metal-induced neurotoxicity

Public health interventions, exposure surveillance, early biomarker detection, and targeted therapeutic strategies are needed to reduce the neurological burden of heavy metal exposure (114). Lead, mercury, arsenic, and cadmium are still common environmental contaminants, and preventive measures should focus on reducing exposure at both the population and individual levels. Environmental risk reduction will remain a critical priority, given the need to improve drinking water quality, control industrial emissions, enhance occupational safety, and reduce contamination of food supplies. The neurotoxic effects of heavy metals were more pronounced in vulnerable populations such as pregnant women, children and older adults. Early detection of exposed persons is essential from a translation point of view to prevent any irreversible neurological damage. Biomonitoring (e.g., measuring heavy metal levels in blood, urine, hair, and nails) can also provide useful information about exposure burden and could assist in risk stratification. The incorporation of exposure biomarkers with markers of oxidative stress, neuro-inflammation, mitochondrial dysfunction, and neurodegeneration can enhance the ability to detect subclinical neural injury before the onset of neurological signs and symptoms (115). The advent of neuroimaging and multi-omics technologies provides additional tools for identifying molecular signatures of heavy-metal-induced neurotoxicity and disease susceptibility. Therapeutic measures are mainly directed toward decreasing the toxic load and minimizing the secondary pathological processes. Chelation therapy is a well-established treatment for severe cases of heavy metal poisoning, but should be used with great caution due to adverse effects and possible deficiency of essential trace elements. Furthermore, there is emerging evidence that antioxidant, anti-inflammatory and mitochondria-targeted therapies can mitigate oxidative damage, maintain cellular bioenergetics, and dampen neuro-inflammatory signaling that occurs as a result of exposure to heavy metals (116). Additional neuroprotective effects might be achieved via nutritional interventions that help maintain gut microbiota homeostasis and endogenous antioxidant defenses. Precision prevention strategies that combine environmental exposure assessment, molecular biomarkers and individualized risk profiles to lower long-term neurological impacts of heavy metal exposure in the future should be emphasized.

## 9 Future directions and research gaps

Future research should adopt a progressive paradigm incorporating longitudinal monitoring, systemic exploration, and systems-level analysis to elucidate how cumulative environmental exposures shape biological susceptibility to neurological disorders across the lifespan (117). The existing evidence indicates that environmental toxicants, lifestyle factors, and ecological conditions affect neural development, age-related changes, and disease susceptibility. Still, most current studies using cross-sectional designs are sensitive to associations rather than causal relationships. Longitudinal strategies and exposome-based approaches thus play a critical role in delineating temporal relationships among exposure timing, dose accumulation, and biological response, because neurological pathology can occur decades after the original environmental harm (118). Multi-omics approaches offer an unprecedented opportunity to characterize the complex biological responses underlying environmentally induced neurological disorders. Rather than focusing on isolated molecular changes, integrated analyses of genomic, epigenomic, transcriptomic, proteomic, metabolomic, and microbiomic datasets can reveal interconnected regulatory networks that drive disease susceptibility and progression. Environmental toxicants may induce epigenetic modifications that alter gene expression, leading to downstream changes in protein function, cellular metabolism, and neuro-immune signaling (119). Simultaneous evaluation of these molecular layers may facilitate the identification of biomarker signatures that more accurately reflect cumulative environmental exposure and biological vulnerability than single biomarkers alone. Recent advances in machine learning and systems biology have further enabled the integration of large-scale multi-omics datasets with environmental exposure histories, clinical phenotypes, and neuroimaging findings. Such approaches may improve predictive modeling, enable earlier diagnosis, and support precision prevention strategies tailored to an individual’s genetic background, exposure profile, and biological risk factors. Ultimately, multi-omics-based biomarker discovery has the potential to transform neurological medicine by enabling the identification of high-risk individuals before irreversible neurodevelopmental or neurodegenerative changes occur. Constant observation of internal and external exposures and repeated biological sampling would enable investigators to trace exposure curves, identify periods of high vulnerability, and distinguish short-term adaptive mechanisms from long-term pathological events (120). Incorporation of multi-omics technologies into systems neuroscience is another key priority, as environmentally induced neural dysfunction is mediated by coordinated changes across genomic, epigenomic, transcriptomic, proteomic, metabolomic, and microbiomic layers rather than independent changes in individual molecules. Regulatory neurons that connect environmental stimuli to cellular signaling, metabolic adaptation, and synaptic regulation can be reconstructed using high-throughput platforms capable of capturing these multidimensional datasets (121). Convergent pathways may then be identified using computational modeling and network-based analytics, which reveal shared mechanistic hubs across neurodevelopmental and neurodegenerative disorders, thereby identifying targets that would not be evident when individual pathways are studied separately. Identification of biomarkers that reflect joint environmental and biological effects is also possible through systems-level integration, thereby enhancing predictive accuracy for disease risk and progression (122). Individual risk assessment and targeted prevention are a new frontier in the field, as there is great diversity in individual genetic background, detoxification efficiency, immune responsiveness, metabolic plasticity, and epigenetic plasticity, all of which influence the extent to which environmental exposures affect neural health. Adding personal genomic data, exposure history, lifestyle, and molecular biomarkers to predictive algorithms would enable early identification of high-risk individuals, before it is too late to prevent neural damage (123). This type of predictive model would enable the application of personalized preventive strategies, such as targeted reduction of exposure, personalized nutritional optimization, microbiome-based therapeutic approaches, and pharmacological adjustment of susceptible molecular pathways. The solution to these research gaps would be interdisciplinary teamwork between neuroscientists, toxicologists, epidemiologists, clinicians, computational biologists, and experts in the field of public health because environmental factors that contribute to brain diseases do so at the molecular, cellular, organismal, and societal levels concomitantly (124). The emerging findings would require cohesive efforts that combine sophisticated analytical tools, population-based studies, and mechanistic testing to achieve clinical significance. Advances in this regard can transform neurological medical practice into a more reactive and proactive science that reduces the impact of diseases through early intervention and informed environmental management (125). Future studies should focus more on understanding the molecular mechanisms underlying the role of heavy metals in neurodegeneration throughout life. While there is evidence that exposure to lead, mercury, arsenic and cadmium is associated with neurological dysfunction, the mechanisms by which chronic environmental exposure leads to progressive neurodegeneration are not fully understood. Specifically, more research is required to understand the interplay among heavy metals, mitochondrial quality control mechanisms, autophagic and proteasomal degradation pathways, and epigenetic networks that regulate neuronal survival and stress resilience. Mechanisms by which heavy metals affect the aggregation and propagation of pathogenic proteins such as amyloid-β, tau, and α-synuclein should also be studied further. Heavy metal exposure appears to cause chronic changes in microglial and astrocytic function that lead to chronic neuro-inflammation, but the time course and reversibility of these changes are still poorly understood (126). The combination of multi-omics approaches, including transcriptomics, proteomics, metabolomics, epigenomics, and sc-omics, could offer additional insights into cell-type-specific responses to environmental toxicants. In addition, there is a need for longitudinal studies that integrate exposure assessment, molecular biomarkers, and neuroimaging measures to determine whether cumulative exposure to heavy metals causally influences disease progression. The potential to identify early markers of neurotoxicity associated with heavy metal exposure and to develop targeted interventions to restore mitochondrial function, reduce oxidative stress, and modulate neuro-inflammatory signaling pathways is promising for future translational research. This type of investigation will play a crucial role in the development of precision prevention strategies and promote understanding of environmentally mediated neurodegenerative diseases (127).

## 10 Conclusion

There is an interdependence between neurodevelopmental and neurodegenerative disorders, as brain vulnerability is influenced by environmental exposures spanning the lifespan, and evidence of converging molecular pathways from prenatal insults, adolescent exposures, and physiological changes related to aging that compromise neuronal stability, synaptic signaling, and neuro-immune regulation. A combination of epidemiological and mechanistic results has shown that pollutants, heavy metals, dietary toxicants, and chemical stressors disrupt mitochondrial function, promote oxidative damage, and degrade protostasis, establishing a biological spectrum that spans early developmental changes to later-life neurodegeneration. The convergent processes of chronic inflammation, impaired autophagy, and cumulative cellular stress can explain how environmental factors do not act independently but rather affect neural resilience over time within the context of genetic vulnerability. This paradigm has been further extended by the realization that the GBA forms a regulatory interface that demonstrates the summation of the microbial metabolite and environmental exposures on neurobiology, immunological signaling and neuronal survival; The current findings have translational implications, exposure responsive biomarkers, risk stratification models and preventive environmental policies can make it possible to detect and manipulate exposure-response relationships at the earliest possible stage, thus preventing the accumulation of damage to neurons in individuals. The future of this area lies in longitudinal exposome studies, multi-omics studies, and precision prevention solutions that, taken together, would shift neurological medicine from a reactive focus to one that secures brain health throughout life.

## Glossary

AD: Alzheimer’s disease
ADHD: attention-deficit/hyperactivity disorder
ALS: amyotrophic lateral sclerosis
ASD: autism spectrum disorder
ATP: adenosine triphosphate
As: arsenic
BBB: blood–brain barrier
Cd: cadmium
CNS: central nervous system
DALYs: disability-adjusted life years
DNA: deoxyribonucleic acid
DOHaD: developmental origins of health and disease
ETC: electron transport chain
GBA: gut-brain axis
Hg: mercury
H₂O₂: hydrogen peroxide
IQ: intelligence quotient
MAPK: mitogen-activated protein kinase
mTOR: mechanistic target of rapamycin
mtDNA: mitochondrial DNA
O₂^−^: superoxide anion
Pb: lead
PD: Parkinson’s disease
ROS: reactive oxygen species
RNA: ribonucleic acid
SCFAs: short-chain fatty acids
UPS: ubiquitin-proteasome system
ZO-1: zonula occludens-1
